# *Ex Vivo* Cytokine mRNA Levels Correlate with Changing Clinical Status of Ethiopian TB Patients and their Contacts Over Time

**DOI:** 10.1371/journal.pone.0001522

**Published:** 2008-01-30

**Authors:** Liya Wassie, Abebech Demissie, Abraham Aseffa, Markos Abebe, Lawrence Yamuah, Hiwot Tilahun, Beyene Petros, Graham Rook, Alimuddin Zumla, Peter Andersen, T. Mark Doherty, for the VACSEL Study Group

**Affiliations:** 1 Armauer Hansen Research Institute, Addis Ababa, Ethiopia; 2 Department of Biology, Addis Ababa University, Addis Ababa, Ethiopia; 3 The Centre for Infectious Diseases and International Health, Windeyer Institute of Medical Sciences, Royal Free and University College Medical School, London, United Kingdom; 4 Department of Infectious Disease Immunology, Statens Serum Institute, Copenhagen, Denmark; Columbia University, United States of America

## Abstract

There is an increasing body of evidence which suggests that IL-4 plays a role in the pathogenesis of TB, but a general consensus on its role remains elusive. We have previously published data from a cohort of Ethiopian TB patients, their contacts, and community controls suggesting that enhanced IL-4 production is associated with infection with *M. tuberculosis*, rather than overt disease and that long-term protection in infected community controls is associated with co-production of the IL-4 antagonist IL-4d2, alongside elevated IL-4. Here, for the first time, we compare data on expression of IFN-γ, IL-4 and IL-4δ2 over time in TB patients and their household contacts. During the follow-up period, the TB patients completed therapy and ceased to display TB-like symptoms. This correlated with a decrease in the relative amount of IL-4 expressed. Over the same period, the clinical status of some of their contacts also changed, with a number developing TB-like symptoms or clinically apparent TB. IL-4 expression was disproportionately increased in this group. The findings support the hypothesis that elevated IL-4 production is generally associated with infection, but that TB disease is associated with a relatively increased expression of IL-4 compared to IFN-γ and IL-4δ2. However, the data also suggest that there are no clear-cut differences between groups: the immune response over time appears to include changes in the expression of IFN-γ, IL-4 and IL-4δ2, and it is the relative, not absolute levels of cytokine expression that are characteristic of clinical status.

## Introduction

Globally, tuberculosis (TB) is responsible for 2–3 million deaths and over 8 million new cases annually [1], with the majority of cases occurring in developing countries, especially in Sub-Saharan Africa [2]. Lack of known correlates of protection to TB has greatly hampered efforts for global control of the disease. Untreated, smear-positive pulmonary TB (PTB) patients are the main source of infection [3], [4] and individuals living in the same household or otherwise in frequent contact with an infectious patient are therefore at a high risk of infection [5]–[7]. However, for reasons as yet unexplained, the majority of these contacts contain the infection without developing full-blown TB and it currently impossible to predict who will and who will not, develop disease. Likewise, while it is known that both TNF-α and IFN-γ are essential for controlling mycobacterial infections [8]–[11], the use of either factor alone as a proxy marker for disease or immunity has been unsatisfactory [12], [13]. The consensus has thus become that protection or susceptibility is a multi-factorial process, resting on the balance of multiple factors. Assessing immune correlates among close household contacts of infectious patients is therefore a pressing issue, but relies on assessing different factors such as exposure, infection, disease and/or protection from TB.

The basic hypothesis to be tested was that TB patients at admission (particularly patients such as those recruited from Butajira and Hossana hospitals in Southern Ethiopia, who typically have quite advanced disease when they are first seen) represented a “failed immune response” which might improve on treatment, whereas infected, but healthy individuals represented a “protective immune response”. Previous reports on this cohort have suggested that elevated IL-4 mRNA expression in household contacts correlates with heightened immune responsiveness to ESAT-6–a good proxy for infection [14]. However, IL-4 mRNA was also somewhat elevated in healthy, infected community controls: the difference being that such individuals also expressed elevated expression of mRNA for IL-4δ2, an IL-4 antagonist and IFN-γ [15], [16]. These specific cytokines were chosen since IFN-γ serves as a useful proxy for Th1 responses, as well as being essential for immunity, while IL-4 is the prototypical Th2 cytokine and has been associated with active TB. IL-4δ2 (a splice variant of IL-4 that appears to antagonize IL-4 action) was included because its expression appears to correlate with protection from the pathological effects of tuberculosis [14]–an effect that may be shared with other IL-4 splice variants [17]. This approach has previously been shown capable of identifying cytokine expression profiles that correlate with either active or latent TB disease [15], [16], [18] but the earlier studies were limited to comparing responses between different clinical groups at a single time point and could only hint at the evolution of the immune response

The goal of this study was therefore to observe what, if any, changes occurred in these key cytokines over time. We collected blood, and purified leukocytes from smear-positive TB patients or their household contacts before the initiation of treatment of the index case. A second sample was taken at the time the index case successfully completed therapy. While there were few clear-cut differences in individual cytokines between household contacts and TB patients at entry to the study, healthy contacts had significantly higher ratios of both IFN-γ and IL-4d2 to IL-4 than TB patients. Interestingly, as we compared the results in TB patients and the contact group at entry and the end of the study, we saw quite distinct trends, with an increased ratio of IL-4 to IFN-γ or IL-4d2 in all cases being associated with a poorer clinical presentation and a decreased ratio being associated with a better outcome.

Nonetheless, it is important to stress that similar underlying trends were detectable in all of the household contacts, suggesting that rather than protection or susceptibility being associated with the development of strongly-polarized Th1/Th2 responses, that all the TB-exposed individuals made a similar suite of responses (with both “Th1” and “Th2” components). The difference between containment of infection and progression to clinical TB therefore appear to depend on successfully balancing the components of these responses.

## Materials and Methods

### Study participants

Two separate cohorts, involving a total of 180 adults were recruited from Hossana and Butajira Hospitals, 230 and 120 km, respectively, southwest of Addis Ababa, Ethiopia. Of the study participants, 76 were newly diagnosed, HIV-, smear-positive pulmonary TB patients (TB) and 104 were close household contacts, who had been living together with the index case for at least 6 months prior to entry to the study. At entry to the study, the contacts healthy (HHC) contacts who were sputum negative, HIV-, asymptomatic and had normal chest X-rays. Blood samples were obtained from all donors at entry to the study and again 6–8 months later. There was one death among the participants in this study (a TB patient), but the cause of death was not identified so this individual was excluded from the analysis. One TB patient failed therapy and was undergoing retreatment at the time of followup–she also was not included in the analysis. However, not all surviving participants could be located or were willing to provide a second blood sample, and due to technical reasons, two collections of blood samples were lost, so at 6–8 months of follow-up, samples from approximately 46% of the original participants were available for analysis-37 TB patients (TB) and 45 healthy contacts (HHC). However, those from whom a second blood sample could not obtained (or for whom the assays could not be done) were still encouraged to attend the clinic for an examination, and this group comprised another 16 TB patients and 33 healthy contacts, for a total retention within the study of 73%. The median age of all participants was 22 years (range 15–62), and 53% of participants were male. Physicians at the hospitals performed a full clinical examination of all participants, with chest X-ray and sputum collection for smear and culture (where sputum could be produced) as previously described [15], [18]. There were no significant differences in the composition of those who fully participated in the followup visit and those who did not, with regard to age, gender, physical condition, clinical diagnosis or place of residence. TST results are not available, as the test is regarded as unreliable in Ethiopia (where a substantial majority of all adults are reactive [19]) and is neither recommended by local health authorities nor routinely performed. All participants were screened for HIV according to National Ministry of Health guidelines with two rapid tests and confirmed with a further ELISA at AHRI [14] and HIV-positive individuals were excluded from the cohort. Pre- and post-test counseling was offered to all participants and HIV positive individuals (n = 2, both TB patients) were referred to the Ethiopia Multi-Sectoral AIDS Program, which provides care and ART. Only adults (15–60 years of age) who had given written informed consent were included in the study and this work was performed under a study protocol approved by the Institutional and National Ethical Review Committees (AHRI/ALERT and NERC).

### Sampling

Blood samples (25 ml) were brought from the study sites to AHRI laboratory at ambient temperature, in 50ml falcon tubes, containing 2% sodium EDTA. Sputum samples were also collected from the study participants for smear microscopy (with Ziehl Neelsen) and culture, following the standard protocols.

### RNA Extraction and RT-PCR

Unstimulated leukocytes were lysed immediately after blood drawing with the RNEASY Blood RNA system (Quiagen, Dusseldorf, Germany) according to the manufacturer's instructions. The mRNA was transcribed into cDNA, using the Omniscript reverse transcription kit (Quiagen, Dusseldorf, Germany) with oligo dT primers, (according to the manufacturer's instructions), the concentration calculated from the optical density using a GeneQuant spectrophotometer (Amersham Biosciences, Amersham, UK) and stored at −20°C until use.

### PCR amplification of cytokine mRNA

PCR was carried out in a total volume of 50 µl with 1 µg of cDNA using the HotStarTaq Master Mix kit (Qiagen, Dusseldorf, Germany) according to the manufacturer's instructions. Primers were designed to span introns so that amplification from genomic DNA should not occur, and this was confirmed by comparing the results from PCR of RNA preparations and the cDNA that was prepared from it. A negative (no template) control was also included in all PCR assays to test for contamination of reagents. PCR products were visualized by running on 1% agarose (Nusieve, FMC, Rockland, ME) gels containing SBYRgreen (Molecular Probes, Eugene, OR) at 1∶10,000 (5 µl in a 50 ml gel) and normalized against the housekeeping gene, β-actin, quantitated against standard curves using the same primers but based on standardized samples containing known copy numbers of cDNA, as previously described [20]. Our own work carried out during this study suggests that β-actin is not an ideal housekeeping gene [21]) but since the intention was to compare samples over time (and the initial samples had been analyzed using β-actin), it was retained throughout to ensure comparability. The PCR conditions and primers used were designed for the project at University College London (UCL) and the number of cycles was optimized for each cytokine (IL-4, IL-4δ2, IFN-γ) as previously described [15]. The fluorescence of bands in the gel under UV transillumination was read using a 12-bit CCD camera (Sensicam, UVP, San Gabriel, CA) and the data analyzed using the supplied Labworks software.

### Statistical analysis

The data obtained are presented as relative expression of the target gene compared to the housekeeping gene β−actin and are shown as medians±range when comparing groups. Comparison of cytokine message levels between different groups was done using one-way ANOVA (nonparametric, Kruskal-Wallis test), whereas changes between baseline and follow-up results (change over time) were analyzed by pairing individual results and then using a paired T-test on the grouped data. A P-value≤0.05 was considered statistically significant in both cases.

## Results

### Assessment of cytokine production at entry to the study of different clinical cohorts using semi-quantitative RT-PCR

**Figure 1:**
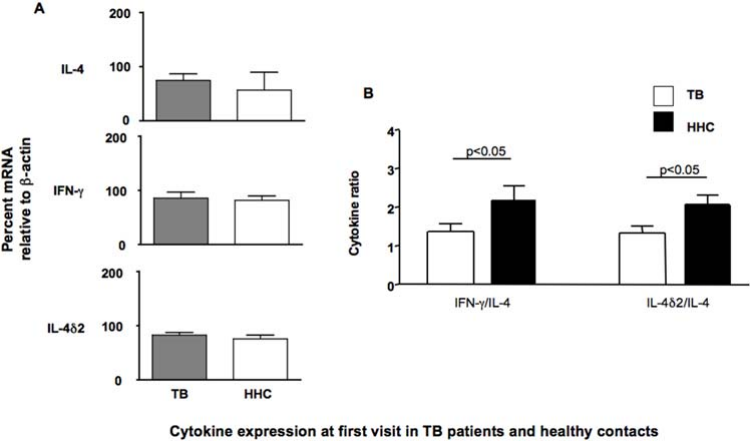
Comparison of relative levels of IL-4, IL-4δ2 and IFN-γ mRNA from unstimulated leukocytes of healthy household contacts (HHC, n = 104) and TB patients (TB, n = 76). Results are presented as medians and ranges of either (A) cytokine message, assessed by RT-PCR, and normalized against β-actin as a housekeeping gene or (B) the ratio of the normalized values of IL-4δ2 and IFN-γ divided by the normalized IL-4 value, as a proxy for the Th1/Th2 balance. Levels of gene expression which were significantly different between groups are indicated, as are the associated p values. Analysis of differences between the groups was performed by Kruskal-Wallis.

Participants were recruited first when sputum-positive TB patients presented at the local TB clinic. Once TB was diagnosed, the index case was asked to return with their household members so that they could also be examined-this is standard practice. If, after counselling and explanation of the study' aims, they were prepared to enter into the study, the adult members of the household were enrolled. At entry to the study, all participants received a clinical examination and a blood draw. Cells from the peripheral blood of each group were lysed (without *in vitro* culture) and mRNA extracted and reverse transcribed into cDNA to analyse the *ex vivo* responses. This cDNA was used for all subsequent analyses. We compared the expression of IL-4, IFN-γ, and IL-4δ2 mRNA in healthy household contacts (designated HHC, n = 104) and index cases (designated TB, n = 76). As seen in Figure 1A, there were no significant differences between these groups when individual cytokines were compared. This is consistent with earlier results (author's unpublished data and [14], [15]). However, there was a weak trend towards higher expression of IL-4 in TB patients and when we compared the ratio of IFN-γ or the IL-4 antagonist IL-4δ2 to IL-4, we saw a significant difference between HHC and TB groups in both cases (Figure 1B). These data suggest that there is a weak bias towards a more Th2-like response in the TB patients compared to healthy contacts when more than one cytokine is considered, but that the use of any single marker is not very informative. This is perhaps not surprising, considering the heterogeneous nature of the groups–the contacts may include individuals in the early stages of TB, those making protective immune responses as well as uninfected individuals–a result confirmed by other analyses [14].

### Assessment of changes in cytokine production over time in different clinical cohorts using semi-quantitative RT-PCR

**Figure 2:**
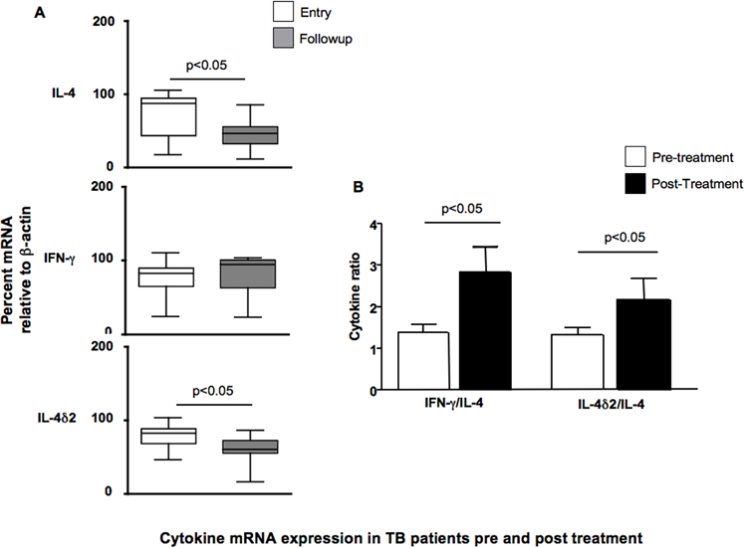
Comparison of relative levels of cytokine mRNA from unstimulated leukocytes from TB patients (n = 37) before and after treatment. Results are presented as medians and ranges of either (A) cytokine message, assessed by RT-PCR, and normalized against β-actin as a housekeeping gene or (B) the ratio of the normalized values of IL-4δ2 and IFN-γ divided by the normalized IL-4 value, as a proxy for the Th1/Th2 balance. Levels of gene expression which were significantly different between groups are indicated, as are the associated p values. Analysis of differences within the groups (over time) was performed by paired t-test.

We attempted to control for some of this variability by investigating the cytokine responses in these groups 6–8 months after entry to the study, by which time a better assessment of their clinical prognosis could be made. At this stage, all the TB patients but one had completed chemotherapy and had become asymptomatic and culture negative, indicative of successful therapy. We assessed cytokine production by PCR as before and as shown in figure 2A, significant declines in mRNA for IL-4 and IL-4δ2 were seen, while IFN-γ levels were essentially unchanged. The steep decline in IL-4 mRNA contributed to a significant increase in both the IFN-γ/IL-4 and IL-4δ2/IL-4 ratios, suggestive of a move towards a more Th1-like response even though IL-4δ2 mRNA also declined somewhat (Figure 2B).

**Figure 3:**
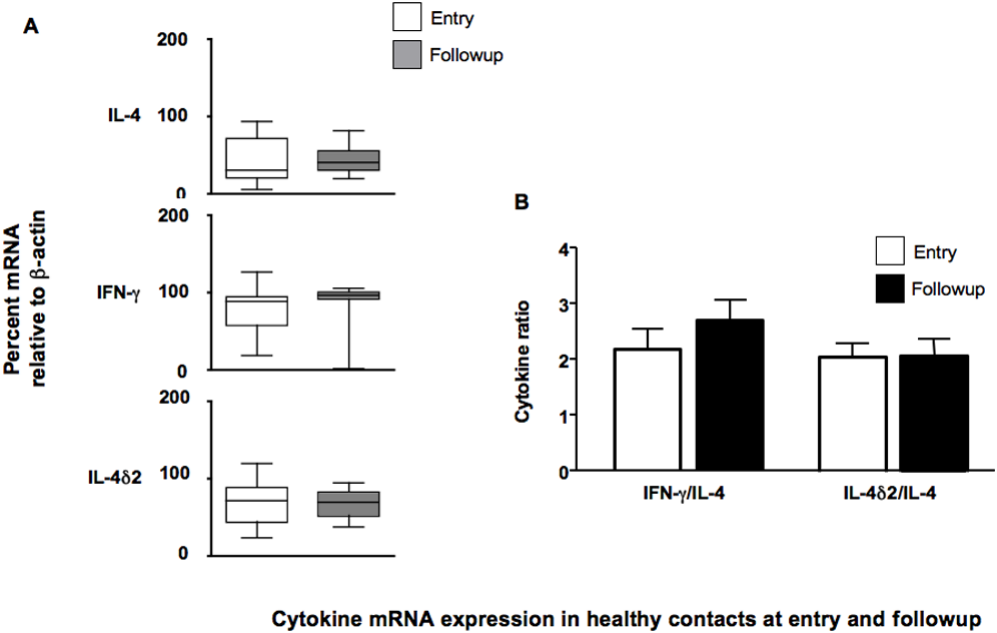
Comparison of relative levels of IL-4, IL-4δ2 and IFN-γ mRNA from unstimulated leukocytes of household contacts who remained symptom-free during the study period (n = 35). Results are presented as medians and ranges of either (A) cytokine message, assessed by RT-PCR, and normalized against β-actin as a housekeeping gene or (B) the ratio of the normalized values of IL-4δ2 and IFN-γ divided by the normalized IL-4 value, as a proxy for the Th1/Th2 balance. Levels of gene expression which were significantly different between groups are indicated, as are the associated p values. Analysis of differences between the groups was performed by Kruskal-Wallis, analysis of differences within the groups was performed by paired t-test.

We performed a similar analysis of cytokine expression in the household contact cohorts, but here the heterogeneous nature of these groups became apparent. Where the inclusion criteria initially designated contacts enrolled as Healthy (HHC–meaning no symptoms or signs of TB) by 6–8 months after entry, some of the contacts had begun to show TB-like symptoms (though they were almost all sputum negative and only a few received a clinical diagnosis of TB (one case of pulmonary TB, 2 cases of extrapulmonary TB). As shown in Figure 3, when we compared immune responses in the contacts who had remained asymptomatic throughout the observation period, there was very little variation in cytokine mRNA expression whether compared singly or as ratios.

**Figure 4:**
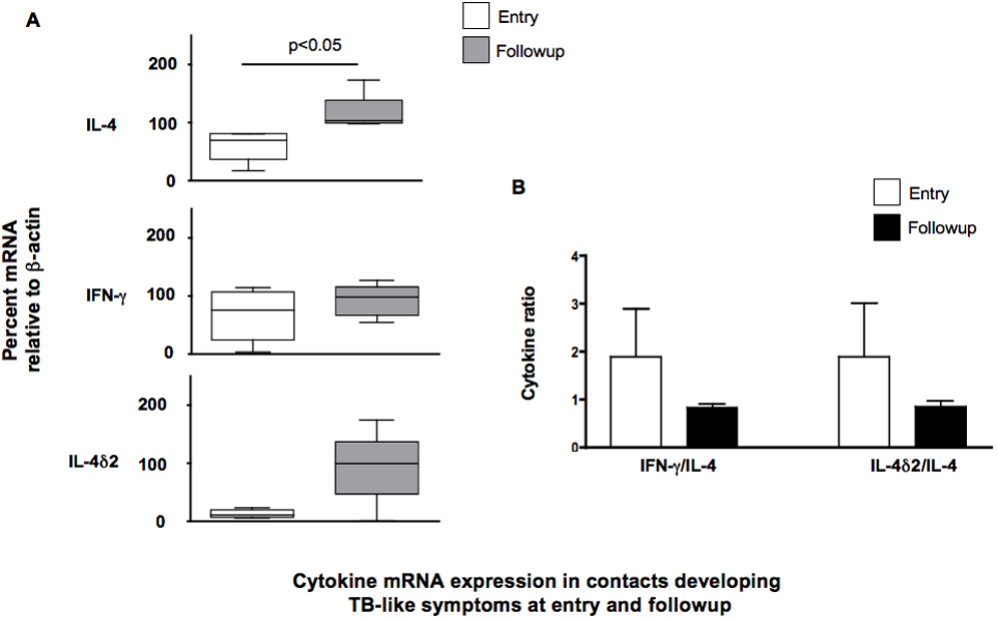
Comparison of relative levels of cytokine mRNA from unstimulated leukocytes of initially healthy household contacts who developed TB-like symptoms during the study period (n = 10). Results are presented as (A) medians and ranges of cytokine message, assessed by RT-PCR, and normalized against β-actin as a housekeeping gene or (B) the ratio of the normalized values of IL-4δ2 and IFN-γ divided by the normalized IL-4 value, as a proxy for the Th1/Th2 balance. Levels of gene expression which were significantly different between groups are indicated, as are the associated p values. Analysis of differences between the groups was performed by Kruskal-Wallis, analysis of differences within the groups was performed by paired t-test.

However, when, we analyzed the data in the cohort who were initially without symptoms but later developed TB-like symptoms (n = 10) a different pattern emerges, as shown in Figure 4. Although the median expression of IFN-γ and IL-4δ2, increased over time, these changes were not significant. This appears to be due to the very variable response, particularly for IL-4δ2: while some showed increased expression of IL-4δ2, nearly half showed the opposite trend. In contrast, all members of this cohort showed an increase in IL-4 mRNA expression leading to a falling ratio of IFN-γ and IL-4δ2 to IL-4 (Figures 4A and 4B).

This means that although the underlying trends for the “Th1” and “Th2” cytokines appear similar, when taken together, they give quite different impressions: the ratio of these cytokines to IL-4 increases in those becoming asymptomatic, and decreases in those developing TB or TB-like symptoms. (Figures 2 and 4).

## Discussion

It is thought that one third of the world's population is latently-infected with *M. tuberculosis*, since only a small proportion of antigen-responsive (and therefore, presumably infected) individuals develop full-blown clinical disease while the majority contain the infection [22]. However, this is not a totally black and white situation: it has been known for many decades that some of those who remain apparently healthy may eventually reactivate their TB infection, while some of those who eventually control the infection may pass through a symptomatic phase. Experience from European sanatoria tells us that even severe disease can persist for years in the absence of chemotherapy–sometimes resolving, more often not [23]. The reason for this differential susceptibility is poorly understood and probably involves multiple innate as well as acquired immune mechanisms. The study of immune responses in *M. tuberculosis*-exposed and possibly infected individuals is therefore essential in understanding the mechanisms at play.

A number of studies both from *in vitro* and *in vivo* experiments and even from *ex vivo* studies have indicated the crucial role of cytokines (particularly the pro-inflammatory cytokines IFN-γ, TNF-α, and IL-12) in TB [24]–[28]. Less information is available on the role of other important mediators of immune function such as IL-4, but what information there is suggests that elevated levels of inflammation-modulating cytokines such as IL-4 and IL-10 are associated with a poorer clinical outcome [29]–[32] or even development of disease [33].

To address this problem, we looked at levels of the prototypical Th1/Th2 cytokines IFN-γ and IL-4, and the IL-4 antagonist, IL-4δ2 in TB patients and their close, household contacts, both at entry to the study (when the index case was first identified) and at the conclusion of treatment of the index case (6–8 months later). In the recruitment area for the study (Southern Nations and Nationalities Peoples Region or SNNPR), almost all admissions are self-referred: thus TB patients are often gravely ill when first seen at the clinic. Additionally, we screened their household contacts and if they agreed to participate, enrolled those who showed no signs of TB or TB-like disease. It should be noted that a substantial proportion of household contacts (n = 58 in this study, or roughly 1/3) were excluded from this analysis because they had already developed TB-like symptoms at the time they were first seen. However, as they met neither the inclusion criteria for HHC (not being healthy), nor for TB (not being sputum positive) they could not be included in the study.

Our clinical studies in Ethiopia have relied on RT-PCR as an adjunct to *in vitro* ELISA and ELISpot, because IL-4 has proven difficult to detect as a protein, being both labile and present in very low quantities [34]. In addition, current antibody pairs cannot differentiate IL-4 and IL-4δ2 ([35] and author's unpublished data). In these studies, plasma was analysed for cytokines by ELISA, but as expected, IL-4 was detected in very few samples (data not shown). There was, however, good correlation between protein levels and mRNA expression for IFN-γ [34].

In the present study, the level of individual cytokines was quite variable and not significantly different, even between TB patients and the healthy contacts (Figure 1). At first glance, this is different from other studies where patients with active TB were shown to have increased numbers of IL-4 secreting T cells [36]–[38], but those studies were done in populations resident in developed countries and typically compare uninfected, non-endemic controls with TB patients. It is therefore not unexpected that results from TB-endemic regions where exposure to many pathogens, including helminthes, is frequent [39] will be different-particularly when examining contacts who often have weeks, if not months, of exposure to a sputum positive index case. And yet, these contacts are the very people whom physicians are most likely to see when considering a diagnosis of TB. As a result we have not used negative controls from developed countries, but have instead assumed that members of a single household will be similarly exposed to pathogens and environmental factors, so that differences in the immune response associated with clinical status (with regard to TB) are most likely related to the TB infection or exposure.

Even taking this limitation into account, a role for IL-4 in the Ethiopian setting is supported by the significant reduction of IL-4 mRNA seen among treated TB patients at follow-up, implying that chemotherapy-induced cure of TB reduced IL-4 production (Figures 2 and 3). This finding is matched by similar, recently published results in treated TB patients from South Africa [40]. The interpretation is further supported by our previously-published work, where TB patients were found to have higher levels of IL-4 than healthy community controls from the same region of Ethiopia [15] but not contacts, and more recent findings that elevated expression of IL-4 among TB contacts in this area correlates with the magnitude of their recognition of ESAT-6 (a proxy marker for the severity of infection [14], [18].

Even more interestingly, that contacts who developed symptoms consistent with TB (persistent cough, haemoptysis, fever, X-ray changes, etc) between the two visits showed a significant increase in the expression of IL-4 mRNA between entry to the study and follow-up, reaching a level equivalent to that seen in untreated TB patients. This suggests that IL-4 expression correlates with some degree to progress to disease (Figure 4). These different outcomes contrast even more starkly when the ratios of cytokine expression are compared–where the appearance of symptoms corresponds to falling ratios of IFN-γ and IL-4δ2 to IL-4, while their disappearance corresponds with the opposite trend. While our inability to prove that all individuals with TB-like symptoms were in fact TB patients means that the data here cannot be conclusive, this assumption is supported by the fact that 3 of these individuals had progressed to full-blown TB at the time of their second visit. At the very least, these observations do support the hypothesis that increased expression of IL-4 (especially compared to other cytokines) in peripheral blood could serve as a surrogate marker for TB-related disease, and that its reduction may be an indicator of restoration of immune response following cure due to treatment.

In the present study, we also measured the message level of IFN-γ *ex vivo* and consistent with our earlier findings, showed that IFN-γ could not discriminate the different cohorts, as its level was comparable in all the study groups [15]. The persistent expression of high levels of mRNA for IFN-γ is not unexpected, given the proinflammatory nature of many surface molecules found on *M. tuberculosis* (for a review, see [41]). Thus, while it is clearly essential for protection from *M. tuberculosis* infection [42]–[44], the level of total IFN-γ alone appears to be of limited value in differentiating between exposure, infection and disease, as reported in both animal [45]–[47] and human studies [48], [49].

However, cytokines do not act in a vacuum and when taken in context, IFN-γ expression can be informative. While it is common practice to describe an immune response as “Th1” or “Th2”, in reality, few, if any, responses are completely polarized to one extreme or the other. It is the balance of cytokines such as IFN-γ and IL-4 (and other modulators such as IL-4δ2, IL-12, IL-23, etc) that determines the outcome of the response. When we compare the ratio of these “Th1-like” and “Th2-like” cytokines, the association between a profile biased toward Th2-like cytokines and clinical disease (or an opposite bias, with continued health) becomes even more pronounced. Similar observations have been made by other groups, showing that a lower IFN-γ/IL-4 mRNA ratio correlated with a more severe disease in TB patients and that this ratio was higher among healthy control subjects [14], [15], [36], [50]. A recent publication suggests that this may be in part due to improved survival of the mRNA for IL-4 compared to its antagonistic splice variant IL-4δ2 in TB patients [38].

In general, the *ex vivo* cytokine mRNA expression patterns we detected seemed to reflect the clinical status of the participants in this study. These observations thus suggest that the higher the IFN-γ/IL-4 mRNA and IL-4δ2/IL-4 ratios, the more likely that *M. tuberculosis* infection will be controlled in the individual. These ratios could therefore serve as valuable markers for disease susceptibility or resistance to TB. However, these patterns cannot be diagnostic of disease or health on their own, particularly since a single determination of cytokine mRNA level in peripheral blood at one moment in time is not adequate by itself as a status marker. More work is needed to establish reliable bounds for cytokine expression, and to validate the assumption that the symptomatic contacts are in fact showing early signs of TB. This work is ongoing.
